# Notes from a field hospital south of Mosul

**DOI:** 10.1186/s12992-018-0346-9

**Published:** 2018-03-06

**Authors:** John M. Quinn V, Omar F. Amouri, Pete Reed

**Affiliations:** 1 Prague Center for Global Health, Institute of Hygiene and Epidemiology, First Faculty of Medicine, Charles University, Studnickova 7, 121 00 Praha, Czech Republic; 2 Al Jamhouri Hospital, Ministry of Health of Iraq, World Health Organization (WHO) Field Hospital, Hammam Al-Alil, Nineveh Province Iraq; 3 Global Response Management (GRM), Erbil, Kurdistan Iraq

**Keywords:** Health security, Tactical combat casualty care (TCCC), Damage control resuscitation (DCR), Damage control surgery (DCS), War and disaster medicine, Northern Iraq, Mosul

## Abstract

This short letter from the field is offered as a rapid communiqué of the emergency medical situation in Mosul and surrounding areas on the eve of the final onslaught to liberate the city. This letter is based on emergency medical work at two World Health Organization (WHO) and Ministry of Health (MoH) Iraq lead Role II+ Field Hospital facilities south of Mosul City from April to June 2017; these facilities are currently and temporarily managed and administered by private medical industry until full handover to MoH Iraq, with WHO support and expert facilitation. The prominence of non-state actors in the conflict, using hybrid warfare tactics that maximize casualties, makes health security a particular challenge for the global community. This challenge requires health leaders and other actors in the region to set clear strategic goals that support public health of the many millions displaced, maimed and affected by the war. Whether in clinical medicine, development, peace and stability operations, or global health diplomacy, the shared values and conviction to best serve vulnerable communities and mitigate morbidity must embrace the lessons of evidenced based practice derived from military medical experience. WHO is leading the charge in disaster response for the conflict in Iraq, and many challenges remain. This might also include developing a new process in emergency medical response that utilizes private contracting to improve efficiency in delivery and overall sustainability.

## Introduction

Fragile and failed nation states compose the majority of present, and likely future, emergency global health crisis. In short, states sit on a spectrum from failure to stable, with increasing fragility pushing states towards failure. Among many factors, institutional strength, transparency, accountability to its citizenry and the ability to protect borders and people and engender an environment for economic, human and health security comprise core metrics in the working thesis of fragile or failed states’ definitions. Ungoverned spaces are loosely described as areas or regions where the state cannot or is unwilling to extend its mandate. A humanitarian disaster attracting current international attention has been the global disorder in the Middle East, notably with the rise of the Islamic State in Iraq and Syria (ISIS)/Islamic State in Iraq and the Levant (ISIL) (sometimes and hereafter referred to as the Islamic State Group (ISG) and/or “Daesh”) with a critical mass of expansion and violence beginning in 2013/2014. The conflicts this has engendered led to a lack of healthcare infrastructure and no institutional support to provide for health or human security that, at is zenith, affected 5 to 7 million people in Northern Iraq, and possibly over 8 million in Syria and bordering regions. Emergency medical services were already unable to cope with the volume and complex needs of the patient population, apart from the medical demands of the growing prevalence of noncommunicable diseases (NCD) in the region.

The World Health Organization (WHO), in close coordination with the Ministry of Health Iraq (Nineveh Province) and Ministry of Health Directorate in Kurdistan (Dohuk and Erbil) provided health security leadership with the establishment and driving of the Health Cluster process for Northern Iraq. This initiative is now in the process of defining indicators for the health response framework to promote sustainable service provision and plans for sustainability. This brings the situation to the current nexus in Mosul and what liberation may mean for health and human security for the city and region and, by implication, what it may mean for the other occupied cities and communities as other liberation efforts are considered for ISG held and occupied cities and regions. A new NATO Office in Baghdad may be able to also support local security services with stability and peace operations in the face of hybrid and asymmetric war to help rid ISG and its partners in violence from Iraq, Syria and the region.

## Health in Mosul post ISG

Islamic State leader Abu Bakr al-Baghdadi declared the caliphate in Mosul in summer 2014, although the beginnings of this terror group can be traced to mid 2003. By fall of 2016, with several cities being liberated from ISG control, thousands of ISG fighters, some with their families, retreated into Mosul. At that time, Mosul City had an estimated local population of anywhere between 1.1–1.6 million civilian population. ISG destroyed many hospital facilities in the city, removed preventative care, moved surgical equipment to Raqqa in Syria, and gutted clinical staffing. Best clinical practices not in keeping with ISG interpretation of Sharia law still occupy and maintain the two main receiving hospitals in the city (as of early June 2017). Specifically, surgical intervention (i.e. laparotomy, enucleation, among others) not entirely clinically indicated may have occurred, and lack of appropriate and timely wound management, debridement, removal of surgical drains, among many others, all lead to sequelae that increase morbidity and mortality of patients. The second author of this short report was originally forced to work in an ISG supported hospital for 2.5 years before escaping to nearby Kurdistan.

### Traumatic injury

The trauma situation in Mosul is severe. Data capture is difficult in war and sometimes incomplete or under-reported. Trauma rates across the Mosul area of operations are rising with over 12,300 reported casualties between October 2016 (the start of military efforts to drive ISG forces out of the city) and 12 May 2017 [1]. It is assumed that a majority of these casualties are from warfighting activities, most notably in western Mosul. Anecdotally a majority of patient are women and children from both sniper, VBIED^1^ and mortar fire; the onslaught from ISG and possibly, to a lesser extent, Coalition Forces. This anecdotal account is supported by World Health Organization (WHO) recent reporting that, over this period, “73% [of trauma victims] were civilians, of which, 28% were children under the age of 15 years and 25% were females.” [1] Indeed, WHO are coordinating data collection across the health spectrum in order to best serve limited resources and support.

In an effort to mitigate trauma morbidity and mortality, the Iraqi Ministry of Health (MoH) partnered with the WHO in November 2016 to establish emergency field hospitals. These were first introduced with humanitarian relief efforts by Médecins Sans Frontières/MSF, the NGO Samaritan’s Purse and other NGOs as emergency relief. These humanitarian groups act semi-independently and augment the healthcare system in this acute phase of the crisis with WHO as a critical partner for success.

However, the sustainability or capacity expansion of the local healthcare system is not their focus. To create a more sustainable system of emergency medical care, and at the request of the Iraqi Ministry of Health (MoH), the WHO contracted a private medical staffing and management firm. These Role II field hospitals are currently and temporarily privately run, and fully supported by WHO, and co-staffed by Iraqi MoH (i.e. doctors and nurses for emergency, surgical and maternity care); other financial donors are also supporting this temporary option. These field hospitals are currently based in Mosul and neighboring governorates, with the intent that they remain after ISG forces are driven out of the area and serve local communities with emergency and primary healthcare needs, with pure Iraqi medical staff and complete MoH sustainable management. This new concept of humanitarian emergency response is a great opportunity for the people of Iraq and for all patients served.

### Prehospital medicine

In a further effort to address the lack of command and control of medical assets working across military and civilian operations, Trauma Stabilization Points (TSPs) were deployed to support battlefield medicine and military operations, with the mandate to serve and treat both combatants and civilians. These TSPs are staffed with volunteers from humanitarian NGOs, and augment the military and civilian medical care at the line of contact with ISG; with additional Casualty Collection Points established to ensure civilians receive adequate medical care. Official front line trauma data over the October 2016 to May 2017 period reported 2479 cases, of which 6% were children under 5, and 11% were female. [1] Unofficial data suggest that the number may be two to threefold higher. Clinical standards followed for war related mechanisms of trauma must be in line with evidence-informed Tactical Combat Casualty Care (TCCC) guidelines, as listed by the National Association of Emergency Medical Technicians (NAEMT). At the peak of operations, they were hampered by a shortage of surgical specialty supplies, biomedical devices, and other trauma supplies that follow TCCC guidelines at some locations, with logistics proving a challenge to a highly specialized clinical and administrative staff at these medical points.

Observational data from patients we have treated at two WHO Field Hospitals indicate that most arrive from these front-line facilities with injuries from small arms effective and indirect fire; Improvised Explosive Devices (IEDs) with large metal fragmentation and small brass/copper BBs; other projectile injuries; and primary, secondary and tertiary blast trauma. Many children have been shot in the head, pregnant women are often shot in the stomach and abdomen by sniper fire, and many patients of all ages have small and large fragmentation injuries and wounds from head to toe, some of which are life altering. The patterns of traumatic wounding lead to crippling injuries hindering ambulation, both acute and chronic mental stress and related pathologies, permanent and temporary paralysis, multiple nerve injuries, hand and foot amputations, loss of vision, loss of eyes, burns throughout more the 15% of total body surface area (BSA) and countless others.

Complications also arise as the evacuation chain for these victims are challenged by urban, suburban and city warfare, where hundreds of thousands of civilians may be caught in the middle of armed conflicts, with no access to emergency services.

Currently, ambulances (supported financially by UN agencies (such as WHO) and humanitarian organizations) operate on an almost medical-taxi basis, lacking medical supplies to treat or investigate/diagnose a patient, or address life-threatening trauma. In most instances, there are no medical personnel attending the ambulances, and the driver has little to no medical training. The treatment norm is high speed and unsafe driving to the nearest or easiest to reach medical facility the driver knows the location of, as opposed to the facility best suited to treat the injuries. During our fieldwork period (March to June 2017) no consistent mobile phone or radio contact was made with any drivers responding to an emergency; an over reliance on personal text messages used sparingly to support with coordination and response is a communication gap. The strategically placed and expertly staffed TSPs, providing in some instances high quality TCCC and Damage Control Resuscitation-level of care, would see a patient, only to then experience a 30–90 min transport time to definitive surgical care. The patient is alone and unattended in the back of an ambulance, and possibly not breathing or dead upon arrival from easily manageable and preventable causes.

Despite our short time in the field and the Mosul conflict period under consideration, a few lessons already arise from our observations. First, military experiences in trauma medicine comprise the best evidence for organization of clinical interventions in instances of civilian casualties caught in conflict crossfire. Thus the two field hospitals where our observations took place followed the protocols of Role II+ military field hospitals, as laid out by the US Department of Defense. [2] These, WHO led facilities provide competent blood product transfusion for trauma victims, Damage Control Resuscitation (DCR) and Damage Control Surgery (DCS) and are purpose built for surgical and medical emergencies. Surgical supplies were sometimes lacking, however, and the lack of effective patient transfers, as noted above, remained problematic.

Prehospital medicine in Iraq at present appears to be non-existent, except for a few manned TSPs, and represents a potentially simple fix in the form of basic training, minimal staff allocation, and adequate over-the-counter equipment for ambulances and other potential clinical outposts. Since the scope of clinical intervention at the WHO field hospitals has shifted from trauma patients to all emergencies, this now includes NCD emergencies; there is an obvious evidence chasm between supplies and needs for such emergencies, which may create delays in appropriate medical care. This includes the ability to provide sufficient quantities of controlled substances for pain management and sedation, and strengthening post-operative and nursing care. Such concerns must be given greater account if and as ISG is driven from the region and some stability returns.

### Humanitarian vs. privately contracted response: A paradigm shift?

Humanitarian response comes in multiple forms and with many different actors. In conflict and disaster settings, many communities rely on humanitarian response for immediate and sometimes long-term support that augments inadequate of often completely absent healthcare and emergency operations. Such humanitarian response can bring stability to the population, while promoting both human and health security. Iraq is no different in these acute and chronic needs, luckily WHO is supporting this process on near herculean terms, on both an emergency and long-term support basis.

This short report cannot unpack and weigh the complexities of the current paradigm of humanitarian response to war, conflict and disaster or provide definitive definitions for some of the topics and challenges discussed. However, the concept of a global body like the WHO contracting out response to private medical providers is relatively new, and results from its experimentation with this approach in Iraq (Mosul) may suggest a shift in how the global community maximizes diminishing medical resources in conflict settings and pushes sustainable clinical best practices for resource poor settings. In short, private provision and medical contractors can offer extremely highly qualified medical and logistical professionals with clear-cut performance goals. Although the cost is high, if the indicators for success are not met, the company loses the contract and another company takes on the job to do the same, if not more stringent, operational and project goals – this is accountable disaster response. The process is as transparent as can be for rapid response and the outcome is hypothesized to be far more efficient, accountable and certainly most sustainable. This model holds the potential to be more efficient in the long run and much more sustainable in bringing health security to the affected populations. Data produced from this conflict may prove the opposite, but discussion of the results will be heated for the humanitarian community for decades.

This is also more likely to be the case when recipient institutions such as the Ministry of Health are acting in concert with the WHO, offering not only best practices but more importantly taking into account UN basic protections, human dimension and needs and best practices. Indeed this is a very different emergency response process than that of other responding NGOs who are accountable to their donors and quarterly newsletters looking for new donations to support their operations and efforts, and that may not have a tight affiliation or accountability to local health ministries, or collaboration with WHO on-the-ground interventions.

We offer this speculation based on our past experiences and recent fieldwork observations over the past decade; we believe it merits consideration and rigorously designed academic evaluation. Indeed, this is revolutionary and the current paradigm may be under needed disruption; clearly more research needs to address this through cases studies and evidenced based practices.

The casualty rate to liberate Mosul was very high. As liberation operations continue throughout Iraq and into 2018, the demands for medical trauma services will be acute. The health impact of immediate fighting in trauma, and the long term and chronic conditions exacerbated by an attacked health infrastructure, remain core concerns. We call on WHO, the Iraqi MoH and all other medical and public health actors in the conflict zones to renew their support for more health system capacity building to include training and supplies for the evacuation chain and damage control surgery, as well as support for prevention of disease and primary healthcare services.

WHOs’ leadership in these areas has thus far been exemplary, but will continue to be needed as conflicts persist in the near future, especially in fragile and failed states and ungoverned spaces. This leadership may provide health security for decades to come in support of well over 25 million Iraqis. Delivering essential basic health services, including vaccination services, medicines and trauma care, remains a top priority for WHO and other health cluster partners in the region.

## Summary

Structures supporting health and health security in Iraq need support. NATO and other security partners may be able to offer security assistance on many of the gaps highlighted in this communication, as it is the only security organization with the skills and breadth to address the multiple complexities faced in Northern Iraq, and certainly in Syria when that battle comes to its zenith. [3] Other security partners, in concert with WHO will be vital in serving the region and its vulnerable populations. Regardless of which actor is supporting the civilian populations and public health infrastructure, policy and planning must extend past security risks alone and recognize the equal importance of ensuring health care for the civilian populations in Syria and Iraq with implications for the entire region. Security leadership by Iraqi forces and the loose amalgamation of militias are involved in liberating cities held by ISG, but larger security conglomerates like NATO may be required to maintain and hold down any peace that is gained in Iraq and throughout the region, making a path for sustained human and health security. Our experiences with public-private medical partnerships described in this communication may be reproducible in other war and conflict stricken areas, and may act as catalysts for this health security prospect.

## Abbreviations

BSA: Body surface area (% of a burn)
DCR: Damage Control Resuscitation
DCS: Damage Control Surgery
ICRC: International Committee for Red Cross Red Crescent
IED: Improvised Explosive Device
IS: Islamic State
ISG: Islamic State Group
ISIL: Islamic State in Iraq and the Levant
ISIS: Islamic State in Iraq and Syria
MoD: Ministry of Defense
MoH: Ministry of Health
NATO: North Atlantic Treaty Organization
NCD: Non communicable disease
VBIED: Vehicle Borne Improvised Explosive Device
WHO: World Health Organization

## Footnotes

1 VBIED: vehicle borne improvised explosive devices
